# Is the MMSE enough for MCI? A narrative review of the usefulness of the MMSE

**DOI:** 10.3389/fpsyg.2025.1727738

**Published:** 2025-11-21

**Authors:** Giorgia D’Ignazio, Leonardo Carlucci, Maria Rita Sergi, Rocco Palumbo, Laura Dattilo, Michela Terrei, Michela Balsamo

**Affiliations:** 1 Department of Psychology, University of G.d’Annunzio, Chieti-Pescara, Chieti, Italy; 2 Department of Human Studies, Arts, Cultural Heritage, Educational Sciences, University of Foggia, Foggia, Italy

**Keywords:** mild cognitive impairment (MCI), Mini-Mental State Examination (MMSE), early detection, cognitive impairment, diagnostic accuracy

## Abstract

**Background:**

Mild Cognitive Impairment (MCI) is a clinical condition characterized by a decline in cognitive functions greater than expected for an individual’s age and educational level, yet not severe enough to significantly interfere with daily activities. Early identification of MCI is crucial for targeted interventions, monitoring symptom progression, and implementing appropriate therapeutic or support strategies. In clinical practice, screening tools are often used that show clear limitations in sensitivity and in their ability to detect mild cognitive alterations.

**Methods:**

Although the Mini-Mental State Examination (MMSE) is among the most widely used tools for initial assessment in non-specialist settings, it presents significant psychometric limitations in identifying MCI. A critical analysis of its effectiveness is therefore essential to guide an informed choice of assessment instruments.

**Results:**

A narrative review of the literature suggests that the MMSE shows reduced sensitivity in detecting MCI, with a tendency to underestimate mild cognitive deficits. Specific cognitive domains are insufficiently captured by the MMSE compared to more sensitive tools.

**Conclusion:**

In light of these limitations, this review highlights the need to adopt alternative cognitive tests for the early detection of cognitive impairment.

## 1 Introduction

In recent years, the increase in life expectancy and the growing need to understand aging-related processes have stimulated intense scientific interest in this phenomenon. Aging can be defined as a set of biological and physiological processes that, over time, progressively reduce an organism’s capacity to maintain homeostasis and increase vulnerability to disease and functional decline. Despite advances in science and technology, aging remains a complex and not yet fully understood process, characterized by a series of physical, sensory, cognitive, and emotional modifications (Kok et al., 2025).

In high-income countries, demographic aging and declining birth rates have reshaped population structures, increasing the prevalence of age-related pathologies such as dementia. Dementia has emerged as a major diagnostic and care challenge requiring enhanced prevention strategies (Italian National Institute of Health, 2024; Monson, 2023). In Italy, where approximately two million people are affected by dementia, promoting neuropsychological well-being requires a biopsychosocial approach, that move beyond age-related stereotypes [e.g., the portrayal of older adults as inherently fragile or/and physically debilitated subjects; Coelho-Junior et al., 2024; Rothermund et al., 2023)].

The aging process displays considerable interindividual heterogeneity in both speed and patterns of manifestation (Li et al., 2025). This variability arises from the complex interplay of genetic, environmental, and psychological factors. Advancing age entails not only physical and sensory changes but also modifications in cognitive functioning. There is, indeed, broad consensus in the literature that aging is associated with decline in certain cognitive domains, including episodic memory, reasoning, and abstract thinking (Handing et al., 2023). However, not all cognitive abilities are compromised: crystallized intelligence, vocabulary, and culturally acquired competencies, typically remain stable or may even improve across the lifespan. Likewise, aspects related to emotional regulation and social intelligence tend to improve with age, along with reduced impulsivity, likely reflecting the accumulation of life experience and compensatory strategies (Carstensen et al., 1999; Isaacowitz and English, 2024; Terracciano et al., 2025).

In recent years, understanding of age-related cognitive decline has undergone important evolution, thanks to theoretical models that more delineate precisely the trajectories of normal and pathological aging. One of the most influential is the framework proposed by Sperling et al. (2011), which conceptualizes Alzheimer’s disease as a pathological continuum comprising three distinct stages: a preclinical phase, during which biological alterations are already present, but no clinically detectable symptoms are observed; an intermediate phase, identifiable as Mild Cognitive Impairment (MCI); and the manifest phase of dementia. This model contrasts with physiological aging, which typically involves a certain reduction in the ability to acquire and retain new information, as well as occasional difficulties recalling names of people or places.

MCI has traditionally been considered an intermediate stage between normal cognitive aging and dementia, with cognitive decline that is not sufficiently severe to interfere with independence in daily life (Petersen et al., 1999). Contrary to what is often hypothesized; however, MCI does not necessarily represent a prodromal phase of Alzheimer’s disease. Rather, it constitutes a mild and early condition of cognitive impairment characterized by specific neurodegenerative, cognitive, and behavioral features that distinguish it from dementia (Lee, 2023; Pezzuti and Rossetti, 2015; Richard and Brayne, 2014).

Once an MCI diagnosis is established, a more detailed clinical classification can guide monitoring and potential intervention strategies. MCI is generally divided into two main subtypes: amnestic (aMCI), when the predominant cognitive deficit involves episodic and/or semantic memory, and non-amnestic (naMCI), if impairment affects other domains, such as attention, language, or executive functions. Each subtype can further be distinguished in “single domain” form, if a single cognitive function is compromised, or “multiple domains,” when simultaneously decline extend across several cognitive areas, including visuoperceptual, linguistic, or executive functioning (Winblad et al., 2004).

### 1.1 Mild cognitive impairment: definition and measurement limitations of the construct

In the literature, there is no agreement on the terminology used to define MCI indicators. The first set operational criteria for MCI was developed by Petersen et al. (1999). According to these criteria (P-MCI), diagnosis requires the presence of a subjective concern about cognitive deterioration, reported by the patients themselves, a reliable informant, or the clinician. The impairment should be objectively documented through standardized neuropsychological testing, demonstrating performance below age- and education-adjusted norms. Although basic activities of daily living are globally preserved, mild difficulties may emerge in more complex instrumental tasks. Importantly, cognitive symptoms should not meet the criteria for dementia diagnosis.

In 2003, an international symposium of experts revised and expanded P-MCI original criteria, acknowledging that MCI can affect cognitive domains beyond memory. This revision led to the formulation of the so-called “Winblad criteria,” which represented a significant conceptual advance in the definition of MCI. Notably, the updated classification introduced clinical subtypes, based on the type and number of affected cognitive functions (Winblad et al., 2004).

In the Diagnostic and Statistical Manual for Mental Disorders, Fifth Edition (DSM-5; American Psychiatric Association, 2013) and its text revision (DSM-5-TR; American Psychiatric Association, 2023), cognitive disorders are classified as Major and Mild Neurocognitive Disorders (NCDs). Major NCD is characterized by significant cognitive decline accompanied by functional impairment, while Mild NCD involves more modest deterioration without loss of autonomy. This latter condition corresponds closely to the clinical concept of MCI, sharing overlapping diagnostic criteria, such as cognitive decline from a previous level of functioning and preservation of daily autonomy (Sachs-Ericsson and Blazer, 2015). The DSM-5-TR updates and specifies etiologies and diagnostic features, while maintaining a dimensional perspective on cognitive disorders, conceptualizing Major and Mild NCDs along a continuum of cognitive and functional deterioration (First et al., 2022).

The main characteristic of NCD is acquired cognitive decline in one or more cognitive domains (criterion A) based on both concern about cognitive functions (expressed by the individual, a reliable informant, or the clinician), and on performance in an objective evaluation below expected levels or demonstrable decline over time. Both subjective concern and objective evidence are required, as they complement each other. When there is exclusive reliance on objective testing, a disorder may not be diagnosed in high-functioning individuals, where “normal” test performance actually represents a meaningful decline, or a disease may be erroneously diagnosed in subjects whose current “low” performance does not represent a change from their norm or is the result of temporary extraneous factors such as fatigue or illness.

Criterion B concerns the individual’s level of independence in daily functioning. In Major NCD, decline is severe enough to interfere with independence, necessitating assistance in previously independent activities. Individuals with Mild NCD have generally preserved independence, although may report subtle interferences with functioning or complaints that activities require more effort or time than in the past. The boundary between Major and Mild NCD is inherently arbitrary, as the two disorders are positioned on a continuum. Determining precise thresholds is therefore challenging and requires a careful behavioral observation, and integration of all available data. When clinical manifestations fall near the diagnostic border, the implications of making a diagnosis must be thoughtfully considered.

According to the National Institute on Aging and Alzheimer’s Association criteria (NIA-AA, 2011), MCI is characterized by observable concern regarding cognitive change, measurable deterioration in one or more cognitive domains (memory, attention, language, executive functions, visuospatial abilities), and functional preservation of daily activities, though complex instrumental activities may be affected. The degree of decline must not meet criteria for dementia. The National Institute on Aging (NIH, 2011) also suggests clinical recommendations to enhance diagnostic accuracy and facilitate differential diagnosis, particularly for cases most likely associated with Alzheimer’s disease.

The criteria described thus far (Petersen, Winblad, NIA-AA, and DSM-5-TR) share several core features: subjective cognitive decline, objective cognitive impairment, relative preservation of daily activities, and absence of dementia. However, these definitions differ in how each of these features is operationalized (Table 1). The use of standardized diagnostic criteria allows not only for the identification of MCI but also for its classification into subtypes, depending on the pattern of impairment detected. The definition of subtypes carries prognostic value, particularly in predicting potential progression toward dementia. However, the literature indicates that MCI does not always represent a prodromal stage of dementia (Yaffe et al., 2006). Individuals diagnosed with MCI may indeed remain stable over time, exhibit cognitive improvement leading to a return to normal function, or progress to dementia.

**Table 1:** Diagnostic criteria for MCI.

Diagnostic Criteria	Petersen criteria (P-MCI, 1999)	Winblad criteria (2004)	NIA-AA (2011)	DSM-5-TR (2022)
Subjective concern	Presence of subjective concern about cognitive decline (as reported by the patient, an informant or a clinician)	Cognitive decline should be reported by the patient, a reliable informant, or the clinician	Concern about a change in cognitive functioning (observed by the patient, a reliable informant, or the clinician)	Concern expressed by the individual, a knowledgeable informant, or the clinician that there has been a mild decline in cognitive function
Objective cognitive impairment	Assessed through standardized neuropsychological tests, with scores below the age- and education-adjusted norm	Neuropsychological testing indicates cognitive impairment relative to what would be expected for the individual’s age and educational level	Evidence of impairment in one or more cognitive domains (memory, attention, language, executive functions, visuospatial abilities)	A modest impairment in cognitive performance, preferably documented by standardized neuropsychological testing or, if unavailable, another quantified clinical assessment
Global functioning	Preservation of basic activities of daily living (ADL) with mild difficulties in more complex instrumental activities of daily living (IADL)	Activities of daily living (ADLs) should be essentially preserved, although mild difficulties in instrumental activities of daily living (IADLs) may be present.	Preservation of daily living functions, with possible difficulties in more complex tasks	Cognitive deficits do not interfere with the individual’s capacity for independence in everyday activities
Absence of dementia	Cognitive impairment does not meet criteria for a dementia syndrome	Cognitive symptoms should not meet diagnostic criteria for dementia and should be less severe than that observed in dementia cases.	The severity of cognitive decline is insufficient for a diagnosis of dementia	Cognitive deficits are not better explained by another mental disorder

The percentage of reversion to normality varies between 14.4 and 55.6% depending on studies (Canevelli et al., 2016; Petersen et al., 2019), but these individuals remain at higher risk for future recurrence of MCI or cognitive deterioration compared to those who have never met criteria for MCI (with an estimated risk between 55 and 65%). Furthermore, up to 37% of people with amnestic-subtype MCI maintain a stable course without progression to dementia (Perri et al., 2007).

There remains debate regarding the predictive role of MCI in the development of dementia. According to Petersen (2003), the subtype that most predisposes to the development of Alzheimer’s dementia is the amnestic one; while according to Summers and Saunders (2012), the stronger predictors of conversion are deficits in visual and verbal episodic memory, short-term memory, working memory, and attentional processes, corresponding to the amnestic multi-domain condition.

Overall, the risk of developing dementia is higher in subjects with MCI compared to cognitively healthy peers. In the two years following diagnosis, the cumulative incidence of dementia among people with MCI over 65 years of age is estimated at 14.9%, with a relative risk (RR) equal to 3.3 for any dementia and 3.0 specifically for Alzheimer’s disease (Petersen et al., 2018).

Even factors not strictly cognitive, such as the comorbid depressive symptoms’, seem to negatively influence prognosis. It is known that alterations in limbic system functioning may affect both attentional and mnemonic processes, as well as emotional regulation. Furthermore, dysfunction of the hypothalamic–pituitary–adrenal (HPA) axis and reduced serotonin availability have been identified as shared pathophysiological mechanisms underlying both cognitive impairment and depression (Mikulska et al., 2021; Schlosser et al., 2011). Depression is frequently associated with deficits in executive functions, consistent with evidence of frontal lobe functioning involvement in depressive conditions. In some cases of MCI, improvement in depressive condition may lead to partial or complete reversibility of observed cognitive deficits (Frau et al., 2025; Yoon et al., 2017).

Recent evidence (Ren et al., 2023) underscores the importance of discriminating MCI from both manifest dementia and age-related cognitive change, using standardized instruments that support differential diagnosis. In clinical practice, MCI diagnosis continues to rely primarily on clinical criteria, consistent with recommendations from the National Institute on Aging and the Alzheimer’s Association (Albert et al., 2011). While research-oriented diagnostic frameworks increasingly incorporate biomarkers from neuroimaging and cerebrospinal fluid analyses, these methods currently remain limited to research contexts (Hazan et al., 2023).

Early identification of MCI can provide patients and families with opportunities for timely care and support, implementation of interventions, and preventive interventions aimed at delaying or mitigating progression toward dementia (Petersen et al., 2014). Standardized cognitive testing represents a fundamental step in the assessment of older adults presenting with subjective cognitive complaints (Cappa et al., 2024). The choice of instruments is influenced by multiple factors, including clinicians’ familiarity with tests, availability of validated translations, and ease of administration (Czerwinski-Alley et al., 2024; Janssen et al., 2017).

Within psychogeriatric settings, the Mini-Mental State Examination (MMSE; Folstein et al., 1975) and the Montreal Cognitive Assessment (MoCA; Nasreddine et al., 2005) remain among the most widely used screening tools. Although extensive evidence highlights the limited sensitivity of the MMSE in detecting MCI, particularly among individuals with high cognitive reserve or atypical impairment profiles (Arevalo-Rodriguez et al., 2015; Tsoi et al., 2015)—it continues to be recommended in most recent national clinical guidelines (Fabrizi et al., 2024; Istituto Superiore di Sanità, 2024).

Despite its broad use, the MMSE presents several psychometric and structural limitations (Fernandes et al., 2021; Limongi et al., 2019; Carnero-Pardo, 2014; Siqueira et al., 2019). To date, no studies have comprehensively examined its psychometric properties in an international context. Therefore, the present narrative review aims to critically analyze the MMSE and its limitations.

The narrative review format was selected because it allows for an integrative and interpretative synthesis of heterogeneous findings, which may not meet the inclusion or comparability criteria required for a systematic review (Ghosh and Choudhury, 2025; Grant and Booth, 2009).

## 2 Materials and methods

The present narrative review (O’Brien et al., 2014) focused on studies published between 2015 and 2025, retrieved from the following scientific databases: PubMed, Scopus, PsyInfo, and Google Scholar. The search strategy employed the following keywords: “MMSE” OR “Mini-Mental State Examination” AND “Validation” OR “Cut-off” OR “Sensitivity” OR “Specificity.”

Full-text articles were included if they met the following criteria: (a) peer-reviewed empirical studies, (b) adult or elderly populations, (c) assessment of the MMSE for detecting Mild Cognitive Impairment (MCI), (d) both qualitative and quantitative studies; (e) written in English and Italian. Exclusion criteria were a) case reports, conference abstracts, dissertations, and (b) studies that combined the MMSE with other instruments, without separate reporting. The selection process consisted of three stages: (1) title and abstract screening, (2) full-text screening, and (3) final eligibility assessment. A total of 2,219 records were initially identified through the databases. After applying restrictions based on language, publication date range (January 2015–December 2025) and population, 779 articles remained. The majority of identified articles were excluded due to duplication (*n* = 350), publication type (e.g., editorials, letters, protocols; *n* = 250) or lack of original data directly relevant to aim of this narrative review (*n* = 125). Following the three-stage screening process (title/abstract screening, full-text review, and final eligibility assessment), 54 studies met the inclusion criteria and were included in the final synthesis.

## 3 Results

### 3.1 Mini-Mental State Examination (MMSE): strengths and limitations

The Mini-Mental State Examination (MMSE) was originally designed as a brief, easily administered clinical tool that could be rapidly interpreted, features that have supported its widespread use in medical practice (Folstein et al., 1975). Notably, the MMSE was developed in 1975 as a general bedside cognitive assessment for hospitalized psychiatric patients, prior to the formal recognition of the MCI construct (Gallegos et al., 2022; Petersen et al., 2014).

The test, which takes approximately 7–10 min to administer, consists of various tasks assessing temporal–spatial orientation, memory, attention and calculation, recall, and language. The maximum overall score is 30.

The MMSE has been translated into over 50 languages and adapted for various settings, including telephone and visual impairment versions (Carnero-Pardo, 2014; Siqueira et al., 2019). In Italy, however, several methodological issues remain concerning its national validation. The normative study by Measso et al. (1993) provided age- and education-adjusted scores based on a large sample (>1,000 participants) from northern Italy (Veneto and Lombardy). This geographic limitation restricts the generalizability of the norms, given Italy’s considerable cultural, linguistic, and educational heterogeneity. Using non-representative normative data can lead to diagnostic misclassification; therefore, it is essential to interpret MMSE scores critically and within an appropriate cultural context.

The MMSE presents critical issues in item configuration and test structure (Carnero-Pardo, 2014).

The test lacks sensitivity to frontal and executive dysfunctions, and only three points out of 30 assess memory, the domain most affected in the early stages of common forms of dementia. Moreover, the delay interval in the recall subtest is not standardized but depends on the time needed to complete intermediate attention-calculation items (Lyness et al., 2014). Another limitation is that the MMSE cannot be administered to illiterate individuals, as it includes tasks requiring reading and writing. It has limited sensitivity in detecting mild but clinically relevant memory impairments, since only three words are used for delayed recall (Benedict and Brandt, 1992; Cullum et al., 1993; Lacy et al., 2015). From a psychometric perspective, the MMSE presents notable heterogeneity in diagnostic accuracy values related to MCI detection, with wide ranges in sensitivity and specificity depending on the cut-off applied (Creavin et al., 2016). A review of nine studies comparing MMSE performance to standardized MCI diagnostic criteria (Istituto Superiore di Sanità, 2024) revealed inconsistent findings: (1) cut-offs of 24–25 yielded sensitivity between 0.17–0.76 and specificity between 0.75–0.96 (Dong et al., 2013; Larner, 2016; Luis et al., 2009; Ravaglia et al., 2005). (2) Cut-offs of 25–26 resulted in sensitivity between 0.06–0.87 and specificity between 0.74–1.00, on an overall sample of 2,805 participants (Biundo et al., 2013; Dong et al., 2013; Mellor et al., 2016; Saxton et al., 2009; Smith et al., 2007; Yu et al., 2012). (3) A single study using a 26–27 cut-off, with 89 participants (Biundo et al., 2013) reported moderate reliability with sensitivity 0.53 and specificity 0.78. (4) Cut-offs between 27–28 showed sensitivity 0.29–0.85 and specificity 0.45–0.92, conducted on 701 participants (Biundo et al., 2013; Luis et al., 2009; Saxton et al., 2009).

Importantly, MMSE scores are strongly influenced by educational level, leading to overestimation of cognitive ability in highly educated individuals and underestimation in those with low education (Pellicer-Espinosa and Díaz-Orueta, 2022). This bias results in false negatives among highly educated individuals and false positives among those with limited schooling (Edmonds et al., 2016).

O'Bryant et al. (2008) demonstrated that among participants with ≥16 years of education, the standard cut-off of 23/24 yielded sensitivity of 66% for dementia and 45% for cognitive impairment, underscoring the test’s limited discriminative capacity in this population.

As evidenced by a study conducted by O'Bryant et al. (2008) evidenced, participants with ≥16 years of education, the standard cut-off of 23/24 yielded sensitivity of 66% for dementia and 45% for cognitive impairment, underscoring the test’s limited discriminative capacity in this population.

Summing up, these data outlines the poor stability of MMSE in diagnostic performance for MCI detection and limited usefulness as a screening tool, especially in prodromal phases, when early identification is crucial for timely clinical intervention. A brief screening test for MCI ideally should prioritize sensitivity over specificity, as false positives can be corrected through further careful evaluation, while false negatives delay accurate diagnosis and intervention (Zhuang et al., 2021). Recent meta-analytic evidence confirms the limited diagnostic accuracy of the MMSE (Karimi et al., 2022).

Regional disparities between northern and southern Italy (Vaccaro et al., 2024) further highlight the need for updated region-specific norms to ensure fair assessment across diverse populations. Sensitivity appears particularly inadequate among individuals with low education or atypical cultural backgrounds—situations not uncommon in Italy (Carlesimo et al., 1995; Crane et al., 2006).

In the Italian population, the psychometric limitations of the MMSE are also relied on socio-cultural and methodological factors. Numerous studies evidenced that the test’s psychometric properties do not fully meet the standards required for reliable clinical use in early MCI detection (Aiello et al., 2022b). Regional disparities between northern and southern Italy (Vaccaro et al., 2024) further highlight the need for updated region-specific norms to ensure fair assessment across diverse Italian regions. These findings highlight the need for updated national standards and region-specific normative data to ensure the accuracy and comparability of assessments across the country. These regional differences underline the importance of developing updated, regionally representative norms, especially considering linguistic and educational variability across Italian regions.

In particular, sensitivity appears particularly inadequate among individuals with low education or atypical cultural backgrounds—situations not uncommon in Italy (Carlesimo et al., 1995; Crane et al., 2006).

Despite the availability of standardized Italian versions, few studies have provided robust validation analyses, including factorial structure, test–retest reliability, and ecological. This gap limits the clinical usability of the MMSE in heterogeneous environments and compromised its diagnostic generalizability (Aiello et al., 2022a). The absence of consistent data on long-term predictive validity of the MMSE in the Italian population, along with limited representation of items sensitive to executive functions —often compromised in prodromal dementia— raises doubts about the MMSE’s suitability as a preferred screening tool for cognitive decline (Santangelo et al., 2015).

In this scenario, the Montreal Cognitive Assessment (MoCA) has been often proposed as a more sensitive alternative for early MCI detection. However, the MoCA also presents some limitations: variability in terms of test–retest reliability at 3 months (0.42 < r < 0.81; Karimi et al., 2022); inconsistent findings on the influence of socio-demographic variables on MoCA score (Apolinario et al., 2018; Borland et al., 2017; Bruijnen et al., 2020; Kopecek et al., 2017; Larouche et al., 2016; Santangelo et al., 2015); limited construct validity since the instrument measures only cognitive factors of MCI (Aiello et al., 2022c); insufficient psychometric research in Italian populations (Aiello et al., 2022b; Aiello et al., 2024); and discrepancy in identifying cut-off points for MCI detection (Bosco et al., 2017).

These findings underscore the urgent need for more sensitive and culturally assessment tools to ensure accurate and early detection of MCI.

## 4 Discussion

In both clinical and research settings, the MMSE remains among the most widely used instruments for evaluating cognitive functions. One of the main limitations of the MMSE lies in its relatively its low sensitivity to subtle cognitive impairments, especially during the early phases of MCI (Mitchell, 2009; Wang et al., 2022). This limitation is partly attributable to the global nature of the test, which lacks sufficiently specific items to assess critical cognitive domains such as episodic memory, executive function, and complex attention—domains often compromised in the initial phases of MCI.

Furthermore, the MMSE is affected by significant floor and ceiling effects, which restrict its usefulness in populations with either very low or very high educational levels (Franco-Marina et al., 2010; Kang et al., 2025). From a psychometric standpoint, these effects limit the MMSE’s sensitivity to MCI detection. Specifically, ceiling effect arise when MCI scores cluster near the maximum, while floor effects reduce the ability to discriminate among individuals with severe dementia.

Another critical issue involves standardization and the influence of socio-demographic variables. Factors such as age, education level, and cultural background can substantially affect MMSE performance, potentially leading to misleading score interpretations. These problems underscore the need for alternative or complementary instruments that are more sensitive, specific, and adaptable across different populations. Additionally, copyright protection restricts the MMSE’s accessibility, making its use potentially burdensome or noncompliant with institutional and regulatory regulations (Feldman and Newman, 2013).

The accumulation of all these weaknesses emphasizes the need for cognitive screening instruments with sound psychometric properties. Although the MoCA compensates for several of the MMSE’ weaknesses and demonstrates improved sensitivity in detecting early cognitive decline, it also presents several drawbacks. The MoCA shows variability in test–retest reliability, inconsistent influence of socio-demographic factors, an exclusive focus only on cognitive domains, insufficient validation in specific populations, and discrepancies in cutoff scores (Fernández et al., 2024). These issues highlight the need to develop and implement new assessment instruments.

Overall, while the MoCA offers higher sensitivity, neither instrument fully satisfies the psychometric and cultural requirements for reliable early detection of MCI. This highlights the need for new, more adaptive tools that can better capture subtle cognitive changes.

Moreover, it is necessary to reconsider the concept of Mild Cognitive Impairment as a monolithic entity, in order to establish diagnostic criteria and evaluative instruments that can reliably distinguish physiological cognitive aging and pathological deterioration processes. Furthermore, such revision should aim to identify those subjects with MCI who are at greater risk of progressing toward Alzheimer’s-type dementia.

An ideal cognitive screening instrument for outpatient settings should possess specific operational and psychometric characteristics. First, administration time should be limited to a few minutes to ensure feasibility in routine medical practice. Furthermore, the instrument should require minimal training for administration, scoring, and interpretation, hereby allowing for its use even by non-specialist professionals. To guarantee fairness and generalizability, screening tools must presents invariance measurements with respect to socio-demographic factors not directly related to cognitive decline, such as age, education, gender, language, and cultural background (Wicherts, 2016). In this regard, the application of latent trait theory or Item Response Theory (IRT; Hattie, 1985) represents a key methodological advance, enabling the assessment of construct invariance through Differential Item Functioning (DIF) analyses. Furthermore, the availability of specific norms stratified by age and education, consistent with standard practice in neuropsychological testing, is fundamental to improve diagnostic accuracy.

Additional desirable features include high acceptability among elderly patients, high sensitivity and specificity, and the ability to discriminate even early phases of cognitive deterioration. Finally, an effective screening instrument should assess both memory and executive functions, that is, the cognitive domains most frequently compromised in common forms of dementia (Corbo and Casagrande, 2022; De Roeck et al., 2019). Fundamental psychometric and methodological standards for cognitive screening instruments, including reliability, validity, and clinical utility, are well established in the literature (Wen et al., 2025).

Finally, this narrative review also has several limitations. Potential selection bias, the lack of a systematic evaluation of study quality, and the inherently subjective nature of study inclusion may affect the generalizability and reproducibility of findings. Future research should therefore complement narrative approaches with more rigorous and systematic methodologies.

## 5 Conclusion

The development of new screening instruments should not only aim to achieve higher sensitivity, specificity, and cultural adaptability, but also encompass emerging cognitive constructs, such as clinical metacognition. Integrating metacognitive dimensions into cognitive screening could provide a more comprehensive understanding of how individuals perceive, monitor, and regulate their cognitive functioning (Nelson, 1996; Schraw and Moshman, 1995). This perspective is particularly relevant for Mild Cognitive Impairment (MCI), where subtle deficits in self-awareness and self-regulation may accompany or even precede measurable cognitive decline.

Limited awareness of one’s own cognitive abilities can compromise self-regulation of cognitive behavior, resulting in ineffective use of compensatory strategies and unsuccessful attempts to manage age-related cognitive deficits. These difficulties may negatively impact on both cognitive performance and overall quality of life (Irak and Çapan, 2018). Conversely, higher metacognitive awareness enables individuals to more accurately appraise their cognitive competence and informs self-efficacy beliefs, thereby shaping their approach to cognitive tasks (Hertzog and Dunlosky, 2011; Irak and Çapan, 2018; McGillivray and Castel, 2011; Siegel and Castel, 2019). Growing empirical evidence supports the association between metacognitive functioning and MCI, showing that individuals with amnestic MCI exhibit impaired metacognitive awareness and reduced self-efficacy in everyday cognitive tasks (Bampa et al., 2023; Bampa et al., 2024). These findings highlighs the importance of systematically assessing metacognitive abilities to enhance early detection and to inform tailored intervention strategies.

Future research should therefore aim to development of standardized tools for the systematic assessment of metacognitive processes, integrating these measures into existing cognitive screening protocols for early MCI detection. Such an approach could refine the early identification of at-risk individuals and inform more personalized preventive strategies.

In addition, the development of computerized and mobile cognitive tools could offer promising opportunities for innovation. Digital platforms—such as CANTAB, NIH Toolbox, BrainCheck, and the Digital Processing Speed Test— allow fine-grained, longitudinal monitoring of cognitive performance (Shimada et al., 2025). These technologies enable repeated assessment, detailed analysis of processing speed and variability, and greater standardization, all of which may facilitate earlier detection of subtle cognitive changes that are not captured by traditional screening instruments (Bonvino et al., 2025; van den Berg et al., 2025).

Integrating both metacognitive and technological dimensions into the development and selection of new cognitive screening instruments could represent a crucial step toward improving the ecological validity, precision, and clinical applicability of cognitive assessment in everyday clinical practice.
